# Sensing the Reducing Power to Determine the Cell Fate: Flavin Redox-Switches in Signal Transduction

**DOI:** 10.1007/s00284-025-04562-x

**Published:** 2025-10-21

**Authors:** Chiara Scribani-Rossi, Simone Angeli, Alessio Paone, Sharon Spizzichino, Federica Di Fonzo, Marzia Arese, Francesca Cutruzzolà, Alessandro Paiardini, Serena Rinaldo

**Affiliations:** Department of Biochemical Sciences “A. Rossi Fanelli”, Sapienza University of Rome, 00185 Rome, Italy

## Abstract

Balancing electron donors and acceptors is crucial for bacterial cell to control both anabolism and catabolism rates and to keep at minimum ROS production and the resulting oxidative stress. Efficiency of nutrients uptake and assimilation is therefore linked to the availability of electron acceptors, to finally re-cycle reduced intracellular electron shuttles belonging to catabolism via respiratory chain. In case of excess reduced electron shuttles (named high reducing power), partially reduced respiratory complexes can be populated, which in turn produce ROS and oxidative stress. This is particularly relevant in the biofilm structure, where the stratified community copes with variable environment characterized by nutrients and oxygen gradients; shortage of electron acceptors found in the deeper layers of bacterial biofilm potentially favors the intracellular increase in reducing power. To avoid this risk, bacterial biofilm promotes extracellular electron flux and re-shapes biofilm morphology to increase the surface in contact with oxygen (i.e., electron acceptors). Sensing the reducing power is therefore a strategy to re-adapt the cellular metabolism to the variation of the environmental conditions, to promote the production of defense systems scavenging ROS and finally to tune bacterial behavior, such as chemotaxis and biofilm morphology. In this review, the role of the flavins in sensing the reducing power via signal transducers is discussed, with a focus on selected examples deeply characterized so far. The possible interference of such sensing activity as a strategy to treat infections and biofilm by means of electroceutical tools, is also discussed as future perspective.

**Supplementary Information:**

The online version contains supplementary material available at 10.1007/s00284-025-04562-x.

## Sensing Reducing Power: Why FAD in the Signal Transduction?

Bacteria oxidize organic and inorganic materials to obtain the chemical energy (ATP) to sustain the multitude of processes behind the cellular homeostasis. Energy needs are deeply linked to environmental sensing and nutrients, with the energy-producing and energy-consuming metabolic pathways finely tuned by their availability [1–4]. Among environmental sensors relevant to energy homeostasis, redox-based transducers guide developmental and behavioral decisions (including chromosome segregation, sporulation, aerotaxis, and social behaviors) [5].

**Fig. 1:**
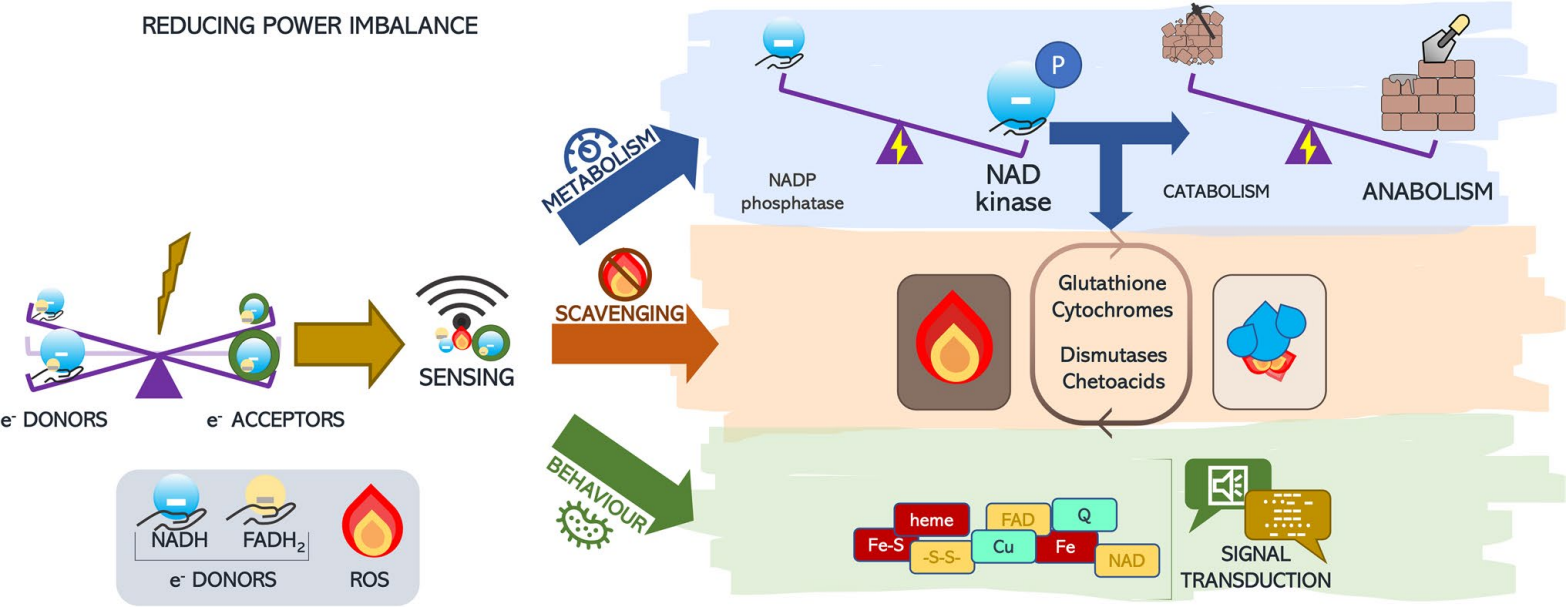
Cellular control of homeostasis under different reducing power conditions. In case of redox insult (flash icon on the left), an imbalance of electron donors/acceptors takes place. The altered reducing power can be directly perceived as the ratio of reduced/oxidized electron donors, leading to (**a**) the tuning of the metabolism via NADPH production (blue pathway) and/or NADH allosteric control of catabolism; (**b**) the scavenging of ROS, come out from imbalanced reducing power; scavengers may take advantage of NADPH accumulation for their re-cycling (orange pathway); (**c**) the re-programming of the cellular behavior via redox-based transducers (green pathway)

Electrons in the cell are stored and shuttled on nucleotide-derived species, in a usable form enough stable to avoid unwanted reactivity; the ratio of reduced *vs* oxidized forms of these electrons’ carriers, namely NADH, FADH_2_ and NADPH, represents the reducing power of the cell. Reaction of excess electrons with oxygen yields ROS (reactive oxygen species) accumulation, which in turn may serve i) as signaling, if their production is contained, and ii) as a trigger for macromolecule damages, if overproduced, due to their uncontrolled reactivity with a plethora of molecules [5, 6] (Fig. 1).

ROS are produced, via superoxide anions, when excess reduced respiratory complexes accumulates, and then “premature” reaction with oxygen takes place [7, 8]. This uncontrolled reactivity may also occur in anaerobiosis, where alternative electron acceptors are used instead of oxygen; in case of N- and S-based acceptors, the overall respiratory process is tuned to ensure donors/acceptors balancing and minimize accumulation of RONS i.e., the Reactive Oxygen, Nitrogen and Sulfur species (the first upon reaction with contaminant oxygen) [9].

It has been proposed that the need to balance the accumulation of donors and acceptors represents a ubiquitous and ancestral “conflict between efficient ATP generation and ROS formation” [7]; moreover, also the nature of the (catabolic) electron sources, i.e., NADH and FADH_2_, should be carefully balanced to avoid a potential source of redox stress. For example, in case of sustained β-oxidation, the FADH_2_/NADH ratio increases, as compared to glycolytic pathway, together with the corresponding flux of electrons to the quinol pool; the potential redox imbalance leads to adaptative response aimed at avoiding a potential stall of Complex I and NADH accumulation (Fig. S1) [10].

In this complex scenario, cell homeostasis is guaranteed by delivering excess electrons to anabolism (Fig. 1 blue path), by scavenging the ROS come out from the respiratory chain (Fig. 1 orange path), by re-directing cell behavior through dedicated signal transduction cascades (Fig. 1 green path).

Redox switch serving one or two component(s) transducers participate in the adaptative response to the reducing power variation; the sensory domain recognizes the cellular redox state and transforms the chemical information into structural re-arrangement to regulate downstream signaling pathways [11]. The sensory domain of these transducers bears redox sensitive moieties such as thiols or cofactors including heme, quinols, iron, copper, iron-sulfur center, flavins and NAD [5, 11–15].

Given the relevance of FADH_2_ in redox homeostasis, a significant number of these proteins have flavins bound to the Per-Arnt-Sim (PAS) domain; these flavin-based redox switches belong to a wider family of sensors including photosensors and voltage responsive regulators [16].

The PAS domain is a conserved structural motif that is ubiquitous in Archaea, Bacteria and Eukarya [17], generally associated with a diverse array of regulatory domains within multidomain proteins serving sensory activity or sustaining protein–protein interaction [18]. Sensory PAS-containing proteins perceive environmental stimuli such as dissolved gases, redox potential or light via redox cofactors (including heme, flavin mononucleotide (FMN), flavin adenine dinucleotide (FAD), 4-hydroxycinnamic acid (4-HCA), carboxylic acids, metals) [19].

Given the multiple redox states of the FAD cofactor, this dinucleotide is useful to finely perceives the reducing power; PAS-containing FAD-based sensors respond to electron availability through the isoalloxazine ring (see below for details) [16] [20]. Only a small number of these specific sensors have been identified as signal transducers namely RmcA, AXDGC2, Aer, NifL, controlling different cellular behaviors such as biofilm morphology, aerotaxis, nitrogen assimilation. A focus on these four transducers is reported as representative examples of one-component systems able to integrate information coming from the environment and the intracellular *milieu*.

## Biofilm Morphology and Reducing Power: AxDGC2 and RmcA

Bacteria can behave as a multicellular community named biofilm, where cells are embedded within an extracellular matrix functioning as scaffold and protective shield. This tridimensional structure leads to a dramatic heterogeneity and shortage of resources availability, including both nutrients and electron acceptors [21].

Environmental stimuli control a metabolic adaptative response to induce biofilm formation or to sustain its maintenance in the different layers of this multifaceted structure [22]; adaptation includes the choice of specific redox pathways and respiration(s) [5], whose effects on biofilm may vary in a strain-specific way [23].

The large part of biofilms gets O_2_ at the interface with air or water, leading to a decreasing gradient in the deeper layers of biofilm; the O_2_ consumption is preferred to alternative respirations, when its reduction is metabolically coupled to electron donors [21].

Biofilm community adapts to imbalance between donors and acceptors by:

- i) Inducing alternative respiratory pathways, such as denitrification in case of N-oxides availability [24];
- ii) Moving electrons in the extracellular space and eventually on biofilm/air interface [25, 26]; carriers such as phenazines may shuttle these excess electrons by taking advantage of the extracellular DNA “cables”, to finally release the high reductive pressure at the bottom of biofilm layer [25];
- iii) Re-shaping of biofilm morphology to yield the wrinkling phenotype; the increased rugosity allows the biofilm community to increase the surface/volume ratio [27] to favor gas exchange with the air interface.

Regulation of phenazine production and wrinkling phenotype are often connected during the adaptative response to redox stress throughout the biofilm, suggesting that a redox-based sensory system orchestrates the adaptation to imbalanced reducing power in biofilm [28] [29].

Environmental control of biofilm architecture is mediated by the intracellular second messenger cyclic diguanylate (c-di-GMP), whose levels may control extracellular matrix production, biofilm morphology and general metabolism on the whole cell or in localized sub-cellular areas [30, 31]. The biosynthesis of this dinucleotide is carried out starting from GTP by dedicated diguanylate cyclases (DGCs), bearing the conserved GGDEF domain. On the other hand, its degradation into the linear form pGpG is catalyzed by phosphodiesterases (PDEs) through the EAL (and, although less representative, the HD-GYP) domain [32]. Genomes and bacteria characterized so far shows a redundancy of GGDEF and/or EAL-containing proteins, since the catalytic portion(s) is often part of multidomain signal transducers (one or two components); the catalytic portion can be made of a tandem GGDEF-EAL moiety, with one of the two serving as allosteric connector in some cases [33].

In the Gram negative *Acetobacter xylinum*, c-di-GMP levels may vary in response to the reducing power [34–36]. In this organism, the biosynthesis and degradation of c-di-GMP are controlled by six homologous proteins sharing the same PAS-GGDEF-EAL architecture, three of them showing DGC activity and the other three showing PDE activity. Among these, AxDGC2 is a DGC which reversibly binds to FAD through its PAS domain; AxDGC2:FAD complex includes a hydrogen bonding network, with Asn94 interacting with the isoalloxazine ring of FAD. The redox state of the FAD cofactor (and not the sole oxygen levels) dramatically affects catalysis: the oxidized form yields a DGC k_cat_ 7.8 folds higher than the reduced form. Mutation of Asn94 abolishes both the FAD binding and the sensitivity to reducing power, further confirming the FAD-mediated switch of the catalytically competent conformation of the enzyme [37].

A link between c-di-GMP, biofilm and reducing power is also found in a more complex one-component system named RmcA from *P. aeruginosa* [38]. RmcA is a multidomain transducers controlling the maintenance of mature biofilm, a phase following the attachment step and community growing. *ΔRmcA* mutant shows, as compared to the wildtype strain, a diverse biofilm growth in the late stages between the 12 to 36 h, depending on the Carbon source. Gene deletion also affects biofilm structure, with the wildtype showing typical “mushroom-like” colonies, while the mutant forming a more uniform layer of cells [39]. Interestingly, RmcA is specifically required under nutrient limitation conditions, suggesting that its c-di-GMP-related activity is linked to nutrient sensing [39]. Moreover, cell-based assays indicate that RmcA controls c-di-GMP consumption in an arginine-dependent manner [40].

The arginine sensing activity is due to its periplasmic domain showing a Venus Fly Trap (VFT) fold, able to specifically recognize environmental arginine [40, 41].

**Fig. 2:**
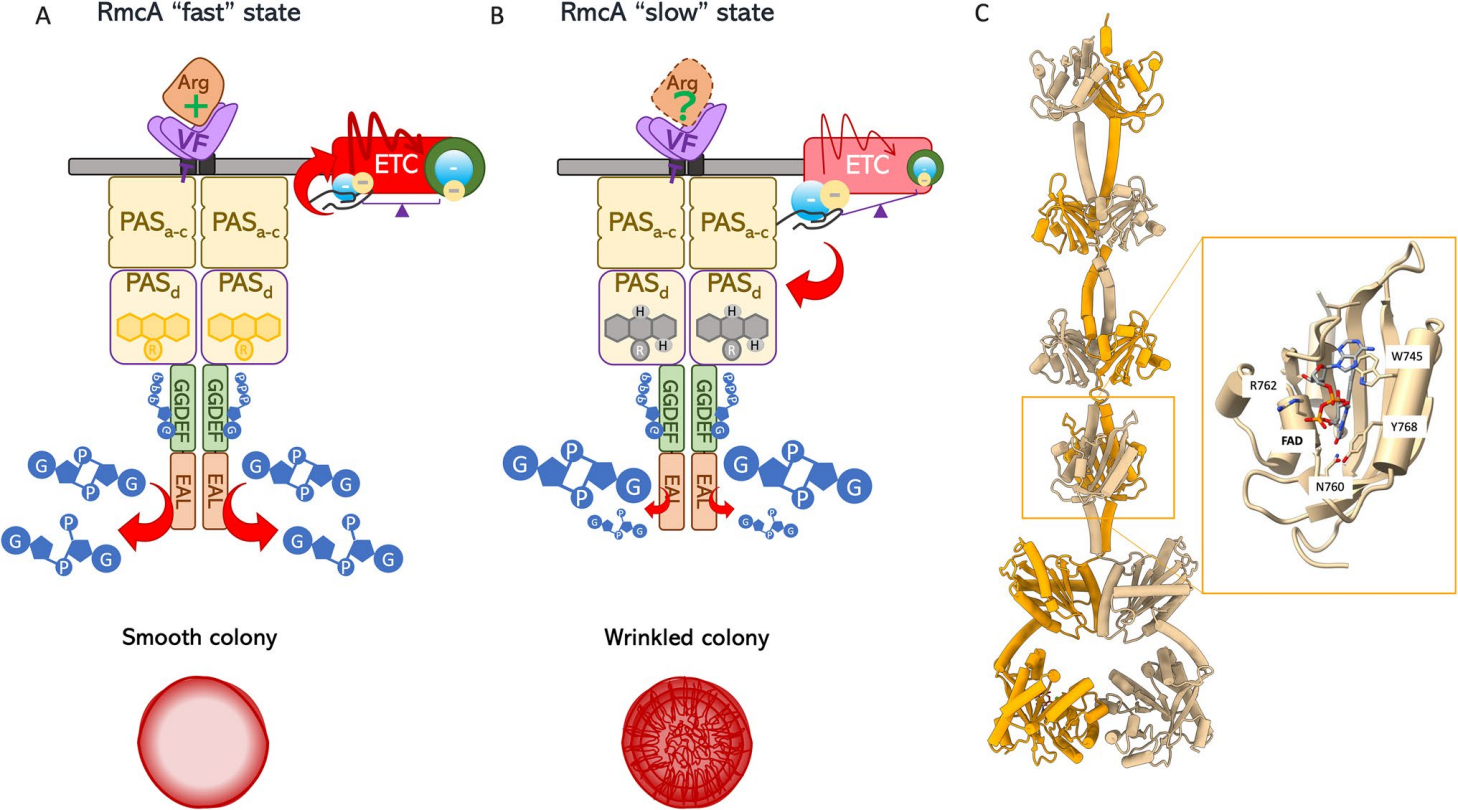
Schematic representation of the redox-dependent regulation of RmcA. (**a**) Under oxidizing conditions, RmcA from *P. aeruginosa* binds to FAD via the PASd domain; in this state the protein is an active phosphodiesterase, controlling c-di-GMP consumption during biofilm maintenance. Indirect evidence indicates that environmental arginine sustains this enzymatic activity, which has been associated to smooth colony phenotype. (**b**) Shortage of electron acceptors, as in the deeper layers of biofilm, leads to increase the reducing power and finally to populate a PASd-FADH_2_ species; this state tunes the downstream phosphodiesterase activity, thus slowing down c-di-GMP consumption. The consequent c-di-GMP accumulation is associated to the rugosity of the biofilm surface (wrinkled colony), which allows bacteria to reach O_2_ with more exposed surface. For both states GTP is depicted, since without this metabolite the enzyme is almost inactive. (**c**) Model of RmcA with the PASd domain in the blow up, according to [38]

Downstream the periplasmic domain, a transmembrane helix links the cytoplasmic portion containing 4 PAS domains (named PASa-d, Fig. 2a) upstream the GGDEF-EAL catalytic portion. The main catalytic activity observed is a GTP-dependent PDE, with the GTP acting as allosteric activator of the EAL domain once bound to the GGDEF one [42].

RmcA is associated to the control of biofilm morphology, since its loss (PA14 *ΔRmcA* strain) leads to a biofilm colony with a hyper-wrinkled phenotype [43]; the wrinkling phenotype is known to be associated to decreased c-di-GMP consumption rate.

A very similar phenotype was observed in a mutant lacking the phenazine biosynthetic pathway (PA14 *Δphz* strain), indicating that both mutations affect biofilm morphology [43]. The superposition of phenotypes between the *ΔRmcA* and *Δphz* mutants suggests that the control of RmcA PDE activity could be associated to redox balance, as already known for phenazine biosynthesis [28].

The redox link between RmcA and PDE turnover was unveiled very recently following the biochemical characterization of the cytoplasmic portion (including the PASa-d and the GGDEF-EAL domains, Fig. 2a). PASd domain binds to FAD and tunes PDE turnover in a redox-dependent manner. Under oxidative conditions, the protein is in a “fast” state where the maximal PDE turnover is observed; on the other hand, under reducing conditions (in the presence of reducing agent and anaerobiosis) a “slow” state is populated and c-di-GMP consumption rate decreases (Fig. 2b) [38]. Interestingly, while electrons, via FAD, reostatically tunes RmcA, GTP acts as on/off switch through the GGDEF domain.

A conserved Asparagine (Asn760) contributes to hydrogen bonding network stabilizing the flavin cofactor (Fig. 2c); upon FAD reduction, a conformational change is predicted to occur affecting the geometry of the contacts and finally the proper dimerization of the downstream GGDEF-EAL domains. Accordingly, the Asn760Ala mutant does not bind to FAD and the PDE activity is insensitive to electrons, showing a pseudo-fast state under all the explored conditions. The same RmcA variant was used to complement the PA14 *ΔRmcA* in a cell-based assay: the Asn760Ala variant not only complements the hyper wrinkled phenotype of PA14 *ΔRmcA* strain, but severely delays the onset of the wrinkles, as compared to wildtype strain [38]. This evidence confirms that the RmcA-mediated c-di-GMP consumption (i.e., PDE activity), which is maximal in the Asn760Ala variant, hampers the wrinkling phenotype. Interesting, this variant also complements the PA14 *Δphz* hyper wrinkled phenotype, further confirming the link between RmcA and phenazines [28] [38]. The FAD/FADH_2_ ratio is related to the reducing power of the cell: once FADH_2_ accumulates, both phenazine and wrinkles are required to decrease electrons accumulation. This requirement is accomplished by decreasing the rate of c-di-GMP consumption due to RmcA PDE activity (favoring the “slow” state and the production of wrinkles); at this stage it is not excluded that other transducers carrying a similar PASd domain are globally controlled by reducing power to explore all the shades of c-di-GMP levels required to direct cell behavior.

## Aer from *Escherichia coli* and Taxis Behavior: Redox Control Of Swimming Motility

Reducing power sensing is also relevant in the motility phenotype, to handle the energy taxis behavior.

Energy taxis is a behavior that allows bacteria to move, in response to sensed stimuli, toward microenvironments with a higher concentration of carbon sources or regions with higher oxygen concentrations [44].

This kind of movement is mediated by methyl-accepting chemotaxis proteins (MCPs) and/or MCP-like proteins; activation of these sensory proteins leads to the autophosphorylation of the core chemotaxis components, named CheA/CheY. The signal transduction machinery upstream CheA/CheY governs chemotaxis (i.e., sensing of environmental chemical composition) or energytaxis (i.e., sensing of internal energetic conditions) [44]. Downstream, CheA/CheY control flagellar rotation; by inverting the direction of flagellar rotation, the trajectory of bacterial movement changes, favoring bacterial approach to (or depart from) the identified signal [44].

When the flagella move counterclockwise, the bacterium moves forward in a straight direction (swimming), while when the flagella move clockwise, the bacterium rotates randomly without advancing (tumbling) [45].

**Fig. 3:**
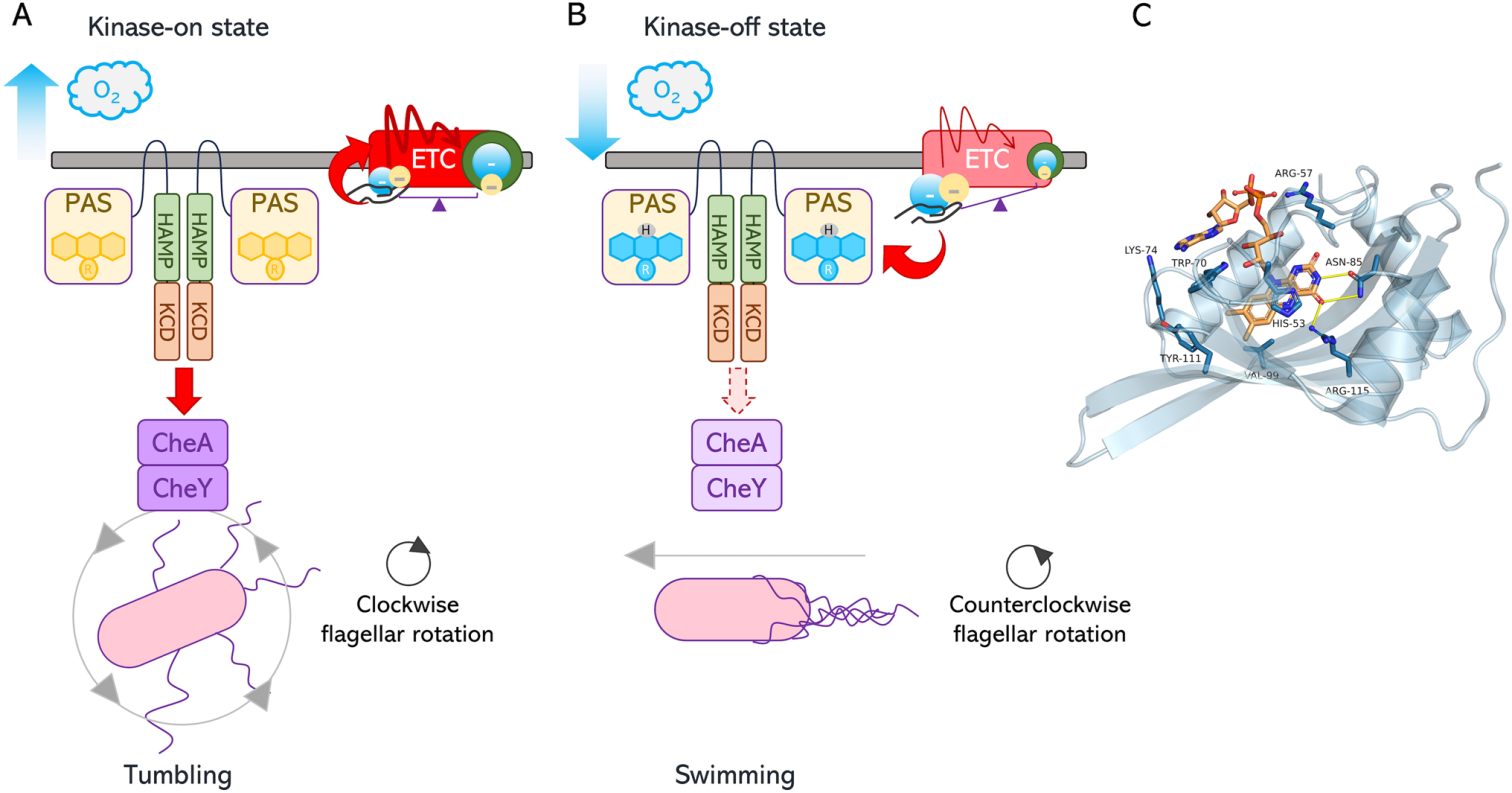
Dependence of Aer activity on O_2_ availability. (**a**) At higher oxygen levels, FAD bound to the PAS domain remains in an oxidized form (kinase-on state), activating the CheA/CheY pathway which promotes clockwise flagellar rotation and cell tumbling. (**b**) At lower oxygen levels, the accumulation of reducing power leads to a reduction of FAD to the anionic semiquinone form or FAD_ASQ_ (kinase-off state), blocking the CheA/CheY pathway and promoting counterclockwise flagellar rotation and smooth swimming. (**c**) PAS domain structure of the Aer from *E. coli* (PDB: 8DIK) represented as cartoons. The key residues interacting with FAD are shown as ball-and-sticks and labeled. The isoalloxazine ring is mainly stabilized by residues Asn85 and Arg155, and via a stacking interaction with His83

In *E. coli*, Aer protein is a sensor of energy levels and determines the movement of the bacterium. This sensor detects changes in oxygen concentration by indirectly controlling the metabolic load of reducing equivalents in the electron transport chain (ETC) [17, 46]. Aer is a dimeric transmembrane chemoreceptor comprised of a cytoplasmic N-terminal PAS domain that binds FAD, a helical transmembrane domain, a cytoplasmic HAMP domain, and a C-terminal coiled-coil kinase control domain (KCD) [47] (Fig. 3a).

The FAD redox state determines the PAS domain re-orientation at level of the interface between the PAS and HAMP domains, to finally control the Aer signaling output; two possible states of the transducer are populated in response to this redox switch, namely the kinase-on and the kinase-off states. The kinase-on state is observed when FAD is completely oxidized (FAD_OX_) and, consequently, the interaction between PAS-HAMP is more stable; under this conformational state, the downstream KCD promotes the autophosphorylation of the histidine kinase CheA and the response regulator CheY, causing the clockwise rotation of the flagella (tumbling) [17, 48–50].

On the other hand, in case of oxygen decrease, FAD yields a semiquinone (FAD_ASQ_), destabilizing the PAS-HAMP interaction: this reorganization leads to a kinase-off state, affecting CheA/CheY activation and promoting counterclockwise rotation of flagella (swimming) [48] (Fig. 3b).

In the binding pocket of PAS domain, residue Arg115 forms a hydrogen bond with N5 of the isoalloxazine ring and this interaction increases with FAD_ASQ_ species, causing a conformational change of the β sheet interacting with the HAMP domain that is essential for signal transmission [51]. Residue His53 is involved in π-π stacking interactions with the isoalloxazine ring. When the Aer flavin is reduced from FAD_OX_ to FAD_ASQ_, the His53 side chain could be positively charged and cause conformational changes in the flavin pocket, similar to the light detection mechanisms characterized by cryptochromes [51, 52]. Positively charged residues of arginine and histidine could optimize the flavin reduction potential causing electrostatic stabilization with the isoalloxazine ring after the reduction.

The Asn85, interacting with the atoms O4 of FAD participates in a network of interactions near the anchor point for the N-terminal α-helix called N-cap relevant to the redox switching, as discussed for the other systems described in this review [51, 53].

This redox-dependent switch directly responds to the intracellular abundance of electrons, whose accumulation is indirectly related to the oxygen availability in the environment; in this way, the bacterium can control its movement with a one-component system to reach a zone where both energy production and redox homeostasis are ensured.

## NifL from *Azotobacter Vinelandii*: Redox-Mediated Transcriptional Control of Nitrogen Fixation

A more integrated system is represented by the NifL protein, a FAD-based redox sensor that regulates nitrogen fixation in the diazotrophic bacterium *Azotobacter vinelandii* [54, 55]. This one-component system integrates the sensing of reducing power reflecting oxygen availability, with the intracellular evaluation of chemical energy (i.e., ATP) and of Carbon *vs* Nitrogen availability.

Nitrogen fixation occurs via nitrogenase, an enzyme able to reduce atmospheric nitrogen into ammonia with the supply of electrons and 16 molecules of ATP. The derived ammonia is assimilated into glutamate (and glutamine) by free-living diazotrophs, like *A. vinelandii.* Therefore, despite the relevance of this reaction (also considering the whole ecological nitrogen cycle), nitrogen fixation needs ATP availability and carbon scaffold(s) to finally yield glutamate/glutamine. Furthermore, nitrogenase is particularly sensitive to oxygen levels as this molecule inhibits its activity [56]. Therefore, genes regulating the expression of nitrogen fixation (*nif*) are tightly regulated in response to oxygen and fixed nitrogen state, to avoid unnecessary energy consumption [57, 58].

Transcription of *nif* genes depends on the transcription factor NifA, whose availability is determined by the aforementioned transducer NifL [59]; environmental conditions hampering nitrogen fixation are perceived by NifL, which traps, via a conformational change, the transcriptional activator NifA in a protein–protein complex [60, 61].

NifL is a multidomain transducer; given the complexity of the multiple sensing activity, an overview of the transduction mechanism is presented, with the structural focus on the redox-active PAS domain discussed separately.

The N-terminal part of NifL contains two N-terminal PAS domains, PAS1 and PAS2. PAS1 binds FAD cofactor and is sensitive to the cellular reducing power, the latter dependent on intracellular oxygen levels [54, 55]; in *K. pneumoniae*, this system is upstream controlled by the oxygen sensor Fnr, which in turn re-program the overall anaerobic metabolism of this bacterium [57, 62].

**Fig. 4:**
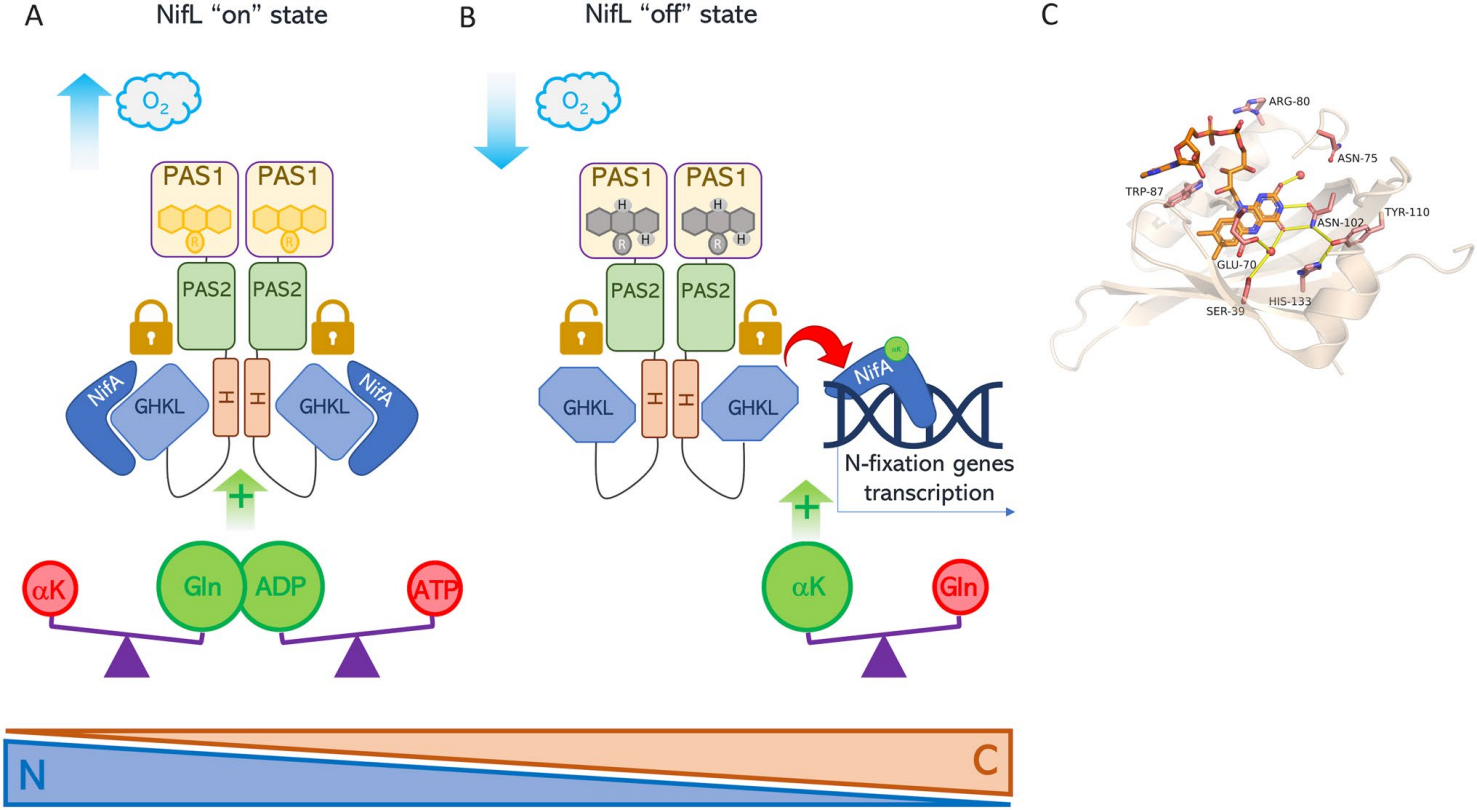
Schematic representation of the conformational changes of NifL. (**a**) Under oxidizing conditions, NifL is in the “on” state with the binding site for NifA exposed; the NifL-NifA complex formation prevents NifA DNA binding (locked), as transcription factor. This complex is stabilized by ADP. This transducer is also regulated by the Carbon/Nitrogen ratio (C and N triangles in the Figure): excess fixed nitrogen (glutamine, Gln in the Figure) with respect to the level of α-ketoglutarate (αK in the Figure) indirectly stabilized the NifL-NifA complex. (**b**) Drop in O_2_ yields FADH_2_ accumulation, which causes a rearrangement of hydrogen bonds in the pocket of the NifL PAS1 domain; this redox-mediated conformational change induces a twisting of the entire protein, assuming an “off” conformation that releases NifA. Free NifA is then competent to act as transcription factor by binding target promoters. Nevertheless, this unlocked state of NifA is sustained by high C/N ratio via αK, which promotes NifA DNA-binding both indirectly and directly. (**c**) Crystal structure of the FAD-containing PAS domain of the protein NifL from *Azotobacter vinelandii* (PDB:2GJ3) represented as cartoons. The key residues interacting with FAD are shown as ball-and-sticks and labeled. The isoalloxazine ring is mainly stabilized by a network of hydrogen-bonds formed by residues Ser39, Glu70, Asn102, Tyr110, His133, and by two structural water molecules (red spheres) present in the FAD-binding cleft

As mentioned above, oxidizing conditions disfavor nitrogen fixation; in the oxidized state, the FAD:PAS1 complex keeps NifL in the “on” conformation, competent to bind to NifA (locked species in Fig. 4a); trapping of NifA by NifL inhibited the transcription of the *nif* genes, thus switching-off the nitrogen fixation.

Conversely, in the absence of oxygen, nitrogenase activity is favored and FADH_2_ accumulates; FADH_2_:PAS1 interaction triggers a rearrangement of the downstream domains (“off” conformation), responsible for NifA release (unlocked state in Fig. 4B) [63]. NifA dissociation from NifL allows this transcription factor to express the *nif* genes, thus switching-on the nitrogen fixation.

The C-terminal portion actively participates in this signal transduction; downstream the PAS1/PAS2 tandem a so-called Q-linker connects to the kinase-like H and GHKL (*in extenso* gyrase, Hsp90, histidine kinase, MutL) domains [64, 65]. GHKL domain integrates the redox PAS-mediated information with the perceiving of the cellular energy and fixed nitrogen status.

The cellular energy status is sensed via the variation of the ADP/ATP ratio [64]: when ADP accumulates, excess ADP binds to NifL, stabilizing the “on” conformation of NifL. This conformation allows NifL to bind to NifA, which in turn cannot activate the transcription of the *nif* genes, thus de facto inhibiting nitrogen fixation.

On the other hand, perceiving of C/N ratio is carried out in response to the intracellular levels of α-ketoglutarate and glutamine (αK and Gln, respectively in Fig. 4), the first serving carbon scaffold for the second. Under excess of fixed nitrogen, Gln accumulates; high levels of Gln indirectly stabilizes NifA:NifL complex (locked in the Fig. 4a) and, as discussed for the ADP sensing, *nif* genes are not transcribed.

In case of low nitrogen, αK accumulates, and low glutamine favors dissociation of NifA from NifL (unlocked in Fig. 4b). The release of NifA allows *nif* genes expression, with NifA requiring αK saturation to activate the transcription of target genes [66].

From a structural point of view, PAS1 contains a central core composed by five-stranded-antiparallel β-sheet that is flanked by N and C-terminal α-helices, like many other PAS domains [67]. The crystal structure of the oxidized form of PAS1 shows that this domain is dimeric, and the two units are asymmetric [59]. In the dimerization interface there is an amphipathic N-terminal α-helix called N-cap [59]. Differently from PAS1, PAS2 doesn’t bind FAD cofactor but change its quaternary structure in response to the redox state of PAS1 and transmit the signal to the downstream domains [63, 68].

In the central core of PAS1, FAD cofactor is in an internal cavity and is involved in a hydrogen bonding network with two internal water molecules and the neighboring amino acids [59]. This network involved the N5 and O4 atoms of the FAD’s isoalloxazine ring and the side chains of residues Ser39, Glu70, Asn102, Tyr110, His133 that line the cavity [59].

Glu70, positioned above the isoalloxazine ring, facilitates reaction of oxygen with the O4 atom of the isoalloxazine ring, under oxidizing conditions [59, 67]. The substitution of the other aforementioned residues with alanine blocks NifL in an “off” conformation, lacking the capability to sense oxygen levels [67].

In the absence of oxygen, the N5 atom of FAD is protonated and the reorganization of hydrogen bonds in the binding pocket is conformationally transmitted from the FAD binding pocket to the downstream domains through the PAS1 dimerization interface [67].

In summary, oxidation of NifL triggers a conformational change that straightens the structure of the protein, exposing the NifA binding interface at the C-terminal portion [64]. While, reducing conditions determine the twisting of the Q-linker which brings the protein into an “off” conformation [64].

## Perspectives and Conclusions

The success of bacterial cell within a community is deeply linked to the control of bioenergetics, to perfectly adapt to environmental conditions. The spatial organization of a community, as in the case of biofilm, requires coping with the shortage of electron acceptors and the subsequent increase of intracellular reducing power [5]. The bioenergetic in that case is dependent on the extracellular electron transfer to reach oxygen or acceptor on the biofilm surface. Nanowires, macromolecules and electron shuttles are responsible for this electrons flux [26], acting as natural “antireductants”.

The reductive stress is a central matter on both biotic systems, to sustain physiological and environmental insults, and abiotic background such as industry, to ensure proper products conservation (including food preservation, textile dyeing stability, deferrization and demanganization) [69]. Understanding the mechanism of reducing power sensing in bacterial background is required to transform the bacterial extracellular electrical conduction into the Achille’s heel of bacteria in chronic infections and pipelines contaminations, to be hit by electroceutical weapons [70]. This approach was proposed as a promising alternative to overused antibacterial agents, to impair *P. aeruginosa* migration and finally decrease wound healing times [71, 72], including the development of a self-charging electroceutical device [73]. The proteins involved in controlling the intracellular reducing power represents promising antibacterial drug targets and vaccine candidates [74] and may be used to develop biosensors, exploitable for multiple biomedical applications [75–77].

Cellular and extracellular bacterial electro-metabolism is studied in synthetic biology to identify strategies for reducing power regeneration and metabolic optimization, to develop optimal *chassis* for redox signaling-driven microbial biosynthesis and biocatalysis [78]. The biotechnological potential of EET tuning touches the bioremediation field, for removal and sensing of toxic minerals and bioenergy production in wastewater treatment [79–81].

Given the multiple fields where bacterial electrons fluxes are involved, understanding the molecular mechanism of redox sensing and behavior direction (Fig. 1 green pathway) is mandatory to take advantage of such a pluripotent phenomenon.

## Supplementary Information

Below is the link to the electronic supplementary material.

Supplementary file1 (DOCX 151 kb)
