# Supplementary material for: Sensing the Reducing Power to Determine the Cell Fate: Flavin Redox-Switches in Signal Transduction
Source: Curr Microbiol. 2025 Oct 21;82(12):563. doi: 10.1007/s00284-025-04562-x (PMC12540587; doi:10.1007/s00284-025-04562-x)
Supplement: Supplementary file 1 — Supplementary file1 (DOCX 151 kb) [file 284_2025_4562_MOESM1_ESM.docx]

**SENSING THE REDUCING POWER TO DETERMINE THE CELL FATE: FLAVIN REDOX-SWITCHES IN SIGNAL TRANSDUCTION**

Chiara Scribani-Rossi#, Simone Angeli#, Alessio Paone, Sharon Spizzichino, Federica Di Fonzo, Marzia Arese, Francesca Cutruzzolà, Alessandro Paiardini#, Serena Rinaldo#*.

Department of Biochemical Sciences “A. Rossi Fanelli”, Sapienza University of Rome, 00185 Rome, Italy.

#These authors contributed equally to the work.

*Corresponding author: serena.rinaldo@uniroma1.it; ORCID 0000-0003-0682-023X. Phone: +390649910713

**SUPPLEMENTARY MATERIAL.**

**
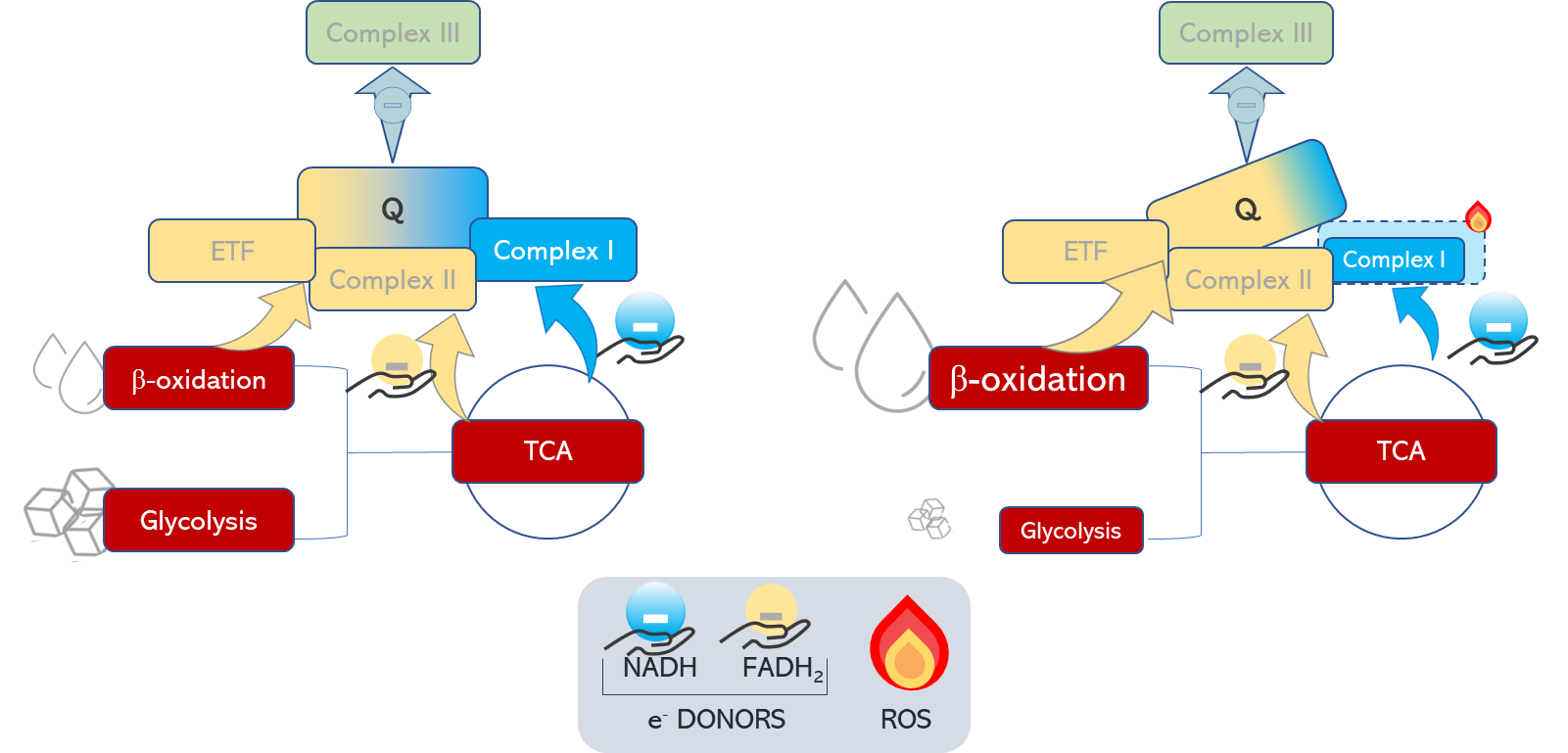
**

**Figure S1. Catabolic pathways sustaining the electron transfer chain.** (Left panel) Glycolysis and β-oxidation pathways oxidize glucose and fatty acids, respectively, yielding NADH and FADH_2_. NADH electrons (including those belonging to β-oxidation, not represented in the Figure for simplicity) are released to Complex I and then to the quinol pool (Q in the Figure); this passage sustains proton pumping. FADH_2_ electrons belonging to TCA Cycle are delivered to the quinol pool *via* Complex II, while those belonging to β-oxidation *via* electron transfer flavoproteins (ETF in the Figure, whose identity depends on the biological background); these passages are uncoupled from proton pumping. Quinols release electrons to Complex III, to sustain the electron transfer chain. (Right panel) In case of mitochondrial sustained β-oxidation, FADH_2_/NADH ratio increases, leading to possible unbalancing of quinols and consequent accumulation of reduced (stalled) Complex I. To avoid this possible source of redox stress, in case of sustained mitochondrial β-oxidation, OXPHOS is tuned, and Complex I degraded [10] (Figure S1, right panel). Bacteria also need to finely balance respiration and β-oxidation to keep at minimum ROS generation, considering that fatty acids are non-fermentable precursors, requiring the presence of an electron acceptor for their catabolism [11]. A recent hypothesis suggested that a high FADH_2_/NADH ratio, as in the case of long chain fatty acids catabolism, should be one of the main sources of the ROS generated by the respiratory chain, which has represented a detrimental aspect particularly during eukaryogenesis [7].
